# A descriptive report of the selenium distribution in tissues from pigs with mulberry heart disease (MHD)

**DOI:** 10.1186/s40813-019-0124-y

**Published:** 2019-09-01

**Authors:** Marianne Oropeza-Moe, Michaela Falk, Marie Vollset, Helene Wisløff, Aksel Bernhoft, Tore Framstad, Brit Salbu

**Affiliations:** 1 Department of Production Animal Clinical Sciences, Norwegian University of Life Sciences, 4325 Sandnes, Norway; 2 Norwegian Veterinary Institute, 4325 Sandnes, Norway; 3 Faculty of Environmental Sciences and Nature Resource Management (MINA)/CERAD CoE, Norwegian University of Life Sciences, 1430 Ås, Norway; 4 Norwegian Veterinary Institute, 0106 Oslo, Norway; 5 Department of Production Animal Clinical Sciences, Norwegian University of Life Sciences, 0454 Oslo, Norway

**Keywords:** Mulberry heart disease, Skeletal muscle, Liver, Cardiac, Muscular, Gastrointestinal, Selenium, Distribution

## Abstract

**Background:**

Mulberry Heart Disease (MHD) is a condition affecting mainly young pigs in excellent body condition. Feed efficient pigs showing high average daily gains are more likely to be affected. MHD has been described as a challenge in Norwegian pig production over the last decade despite abundant supplies of vitamin E, and selenium (Se) close to the upper limits set by the EU. From 2015 to 2017, samples from documented MHD field cases were collected and compared with controls regarding post mortem findings and Se concentrations in numerous internal and external organs were determined in order to characterize the Se distribution, and to identify any differences between MHD cases and controls.

**Case presentation:**

Eight MHD cases from commercial farms and a pet pig producer located in the South West and East of Norway, and three control animals originating from these farms were included in this study. MHD cases and controls were weaned pigs with an average bodyweight (BW) of 17 kg (range 9 to 46 kg BW), with the exception of one pet piglet (Mangalica, 6 kg BW) that had only received sow milk. Selenium was determined in samples from the cardiovascular, digestive, immune, endocrine, integumentary, muscular, respiratory and urinary systems using inductively coupled plasma mass spectrometry (QQQ ICP-MS). All pigs with MHD suffered sudden deaths. Control animals were euthanized without being bled prior to necropsy and sampling. Significantly different mean Se concentrations between MHD cases and controls were found in cardiac samples as well as almost all skeletal muscles (*P* < 0.05)*.* Based on the samples from ten different muscles (except the cardiac samples), mean Se concentrations in MHD cases were 0.34 (0.01) mg/ kg DM compared with 0.65 (0.02) mg/ kg DM in control pigs (*P* < 0.0001). In cardiac samples, mean Se concentrations from MHD cases were 0.87 (0.02) mg/ kg DM vs. 1.12 (0.04) mg/ kg DM (*P* < 0.0001). Additionally, significantly lower Se concentrations compared with controls were found in the liver as well as the caecum, duodenum, gastric ventricle, jejunum, kidney, skin and thymus samples.

**Conclusions:**

Based on the present work, the current common practice regarding tissue analyses in MHD cases could be refined to include other organs than liver and heart. The evident differences in mean Se concentrations in 9 out of 10 samples from the muscular system, could make such samples relevant for complementary measurements of Se concentrations to help confirm the MHD diagnosis. We find it interesting that although our limited number of sampled pigs are different in terms of genetics, size and feeding regimes, the variation of Se concentrations in a given organ was low between MHD cases. Since this report includes a limited number of MHD cases and controls, our results should be corroborated by a controlled, larger study.

## Background

Selenium (Se) deficiency involved in fatal cardiomyopathy is well known in pigs [1–3]. Se deficiency probably causes uncompensated oxidative stress leading to cellular damage, often resulting in death [3]. The importance of Se and selenoproteins in muscle tissue physiology is well documented. Studies have shown that the gene expression, plasma and tissue concentrations of selenoprotein W and selenoprotein P as well as glutathione peroxidase activity, are higher in Se-supplemented than Se-deficient animals [4–8].

Mulberry heart disease (MHD) is a peracute to acute condition, appearing mainly in pigs of two to four months of age. It has, however, been observed in pigs as young as three weeks. Typically pigs in excellent body condition are found dead. The principal gross lesions in pigs succumbing to MHD are straw-colored fluid in the pleural cavity, transudate with fibrin in the pericardium and edematous lungs. The myocardium appears mottled due to transmural hemorrhage and pale necrotic areas. Oxidative stress causes oxidative modifications of myofilament proteins like actin, titin and myosin and can thereby impair the contractility of myocytes [9]. The above mentioned alterations in myocytes combined with macroscopic and microscopic lesions of MHD cases strongly suggest ventricular dysrhythmia followed by acute heart failure. The diagnosis of MHD can be confirmed when the following microscopic heart lesions are observed: In acute cases interstitial hemorrhage is the main lesion, whereas in less acute cases degeneration and necrosis of myofibers, sometimes with mineralization, are observed. [10].

Tremendous advancement within molecular biology and genomics over the last decades, e.g. the sequencing of the porcine genome [11], has facilitated development and improvement of sophisticated research methods and technology. The added value of DNA information to breeding values can now contribute to rapid genetic progress [12]. The breeding goals of most pig breeding companies globally include lean growth efficiency, reduced feed intake per kg growth and reduced backfat [13–17].

The number of publications on MHD and Se concentrations in porcine tissues in peer reviewed literature is limited, and the majority of the existing reports are more than fifteen years old [10, 18–21]. Some of these reports have stated that tissue concentrations of Se in heart and liver samples are within the normal range in MHD cases [10, 18, 19]. Unpublished cases from Norway over the last five years support these statements, also with results based on Se concentrations in liver samples. Nevertheless MHD cases cease to occur in affected herds after Se supplementation, either by injection treatments with Se or feed additives containing Se and vitamin E.

This study was carried out on MHD cases and control pigs reared under field conditions, in order to characterize the Se distribution in multiple tissues of MHD cases and controls prior to and after weaning. To the authors’ knowledge, this is the first report to describe the Se distribution in numerous internal and external organs of pigs diagnosed with MHD.

## Case presentation

The pigs included in this study were submitted for post mortem examinations to the Norwegian Veterinary Institute in Oslo and Sandnes and the Norwegian University of Life Sciences in Sandnes between 2015 and 2017. The pigs originated from five commercial piglet producing farms (Farms 1 to 5) and one pet pig producer (Farm 6) located in the counties of Oppland and Rogaland in Norway (Table 1). The piglet from the latter farm had only been fed sows’ milk.

**Table 1:** Description of included Mulberry Heart Disease (MHD) and control pigs in the case study

No.	MHD/ Control	Farm	Sex	Age (weeks)	Bodyweight (kg)	Genetics	Iron treatment*	Sodium selenite (mg/ kg)	Alpha-tocopherol (mg/ kg)
1	MHD	1	C	14	45.0	HHZL	Oral paste/ iron-enriched peat	0.40	175.00
2	MHD	2	F	6	12.0	HHZL	Injection	0.39	180.00
3	MHD	2	C	6	16.0	HHZL	Injection	0.39	180.00
4	MHD	3	C	6	11.0	LLLL	Injection	0.33	100.00
5	MHD	3	F	6	10.0	LLLL	Injection	0.33	100.00
6	MHD	4	C	6	9.0	DDLL	Oral paste/ iron-enriched peat	0.40	162.00
7	MHD	5	C	8	14.0	DDZL	Oral paste/ iron-enriched peat	0.33	100.00
8	MHD	6	F	6	6.0	Mangalica	Injection	Sow milk	
1	Control	1	C	14	42.0	HHZL	Iron paste and	0.40	175.00
2	Control	2	C	6	18.0	HHZL	Iron injection	0.39	180.00
3	Control	2	F	7	25.0	HHZL	Iron injection	0.39	180.00

Female (F) and castrated (C) pigs in this study originated from five commercial pig producing farms and one pet farmer. The pigs included in this study were both male and female pigs between six and fourteen weeks old. The Se source used in the complete feed was sodium selenite (NaSe) and added levels of NaSe were between 0.33 and 0.40 mg/ kg feed. The vitamin E (vit E) source was alpha tocopherol and added levels were between 100.00 and 200.00 mg/ kg feed. The grower-finisher pigs from Farm 1 were fed liquid feed in combination with whey from approximately 30 kg bodyweight or approximately ten weeks of age. *Iron treatment of the piglets was applied within the first four days of life, either by injecting 200 mg subcutaneously or providing 300 mg of bioavailable iron per os combined with iron-enriched peat until weaning

The Se added to the compound feedingstuffs provided at the farms was sodium selenite at levels between 0.33 mg/ kg and 0.40 mg/ kg feed. The amount of alpha-tocopherol (vit E) added to the feed was between 100 mg/ kg to 200 mg/kg feed. Non-supplemented raw materials used to produce compound feedingstuffs for pigs with Norwegian origin contain negligible Se levels around 0.05 mg/ kg [4]. The feed composition of the feed provided at the different farms is listed in Table 2.

**Table 2:** Feed composition

%	Farm 1*		Farm 2		Farm 3	Farm 4	Farm 5	Farm 6
	MHD1	C1	MHD2/ MHD3	C2/ C3	MHD4 MHD5	MHD6	MHD7	MHD8
Fishmeal LT-94			7.00	7.00		5.00		Sow milk
Barley	35.48	32.16	16.80	10.00	43.13	22.60	27.71	
Soybean cake flour						10.00	8.00	
Soybean meal	13.28	13.21	6.00	4.00	14.20	1.60		
Soybean meal Hipro				6.00			11.00	
Soybean oil	0.38		0.60	1.40	1.00	0.50	1.00	
Wheat	5.00	6.00	50.24	45.71	10.00	49.89	15.00	
Rapeseed cake	8.00	8.00			8.00			
Oats	25.00	30.00	1.70	3.00	9.50		15.00	
Pea starch	5.00		3.00	3.00	5.00		6.20	
Limestone	1.27	1.29						
Animal fat	2.00	4.03	3.20	2.10	3.00	0.70	3.00	
Field beans	5.00	2.00	3.00	3.00		5.00	7.00	
Molasses sugar cane			0.70	0.50	2.00	1.00	2.00	
Sunflower cake			2.30	0.60				
Corn gluten			2.00	0.80				
Corn grits				10.00				
Mikromin Pig^a^	0.23	0.23						
Vitamin ADKB^b^	0.08	0.08	0.08	0.08	0.06	0.08	0.06	
mg/ kg								
Sodium selenite (NaSe)	0.40	0.40	0.39	0.39	0.33	0.40	0.33	
Alpha-tocopherol	175.00	175.00	180.00	180.00	100.00	162.00	100.00	

The feed composition of the feed provided both MHD cases and controls is listed

^a,b^ Additives containing the following per kilogram of diet:

^a^ Fe 96 mg; Cu 20.8 mg; Mn 48 mg; Zn 96 mg; I 0.48 mg

^b^Vitamin A 5700 IU; Vitamin D 1200 IU; Vitamin E

100 mg; Vitamin K 3.72 mg; Vitamin B1 2.4 mg; Vitamin B2

4.5 mg; Vitamin B5 12.0 mg; Vitamin B6 7.2 mg; Vitamin

B12 0.012 mg; Folic acid 1.8 mg; Biotin 0.24 mg

*At Farm 1, the feed was supplemented with whey, accounting for 25% of the total energy

**Fig. 1:**
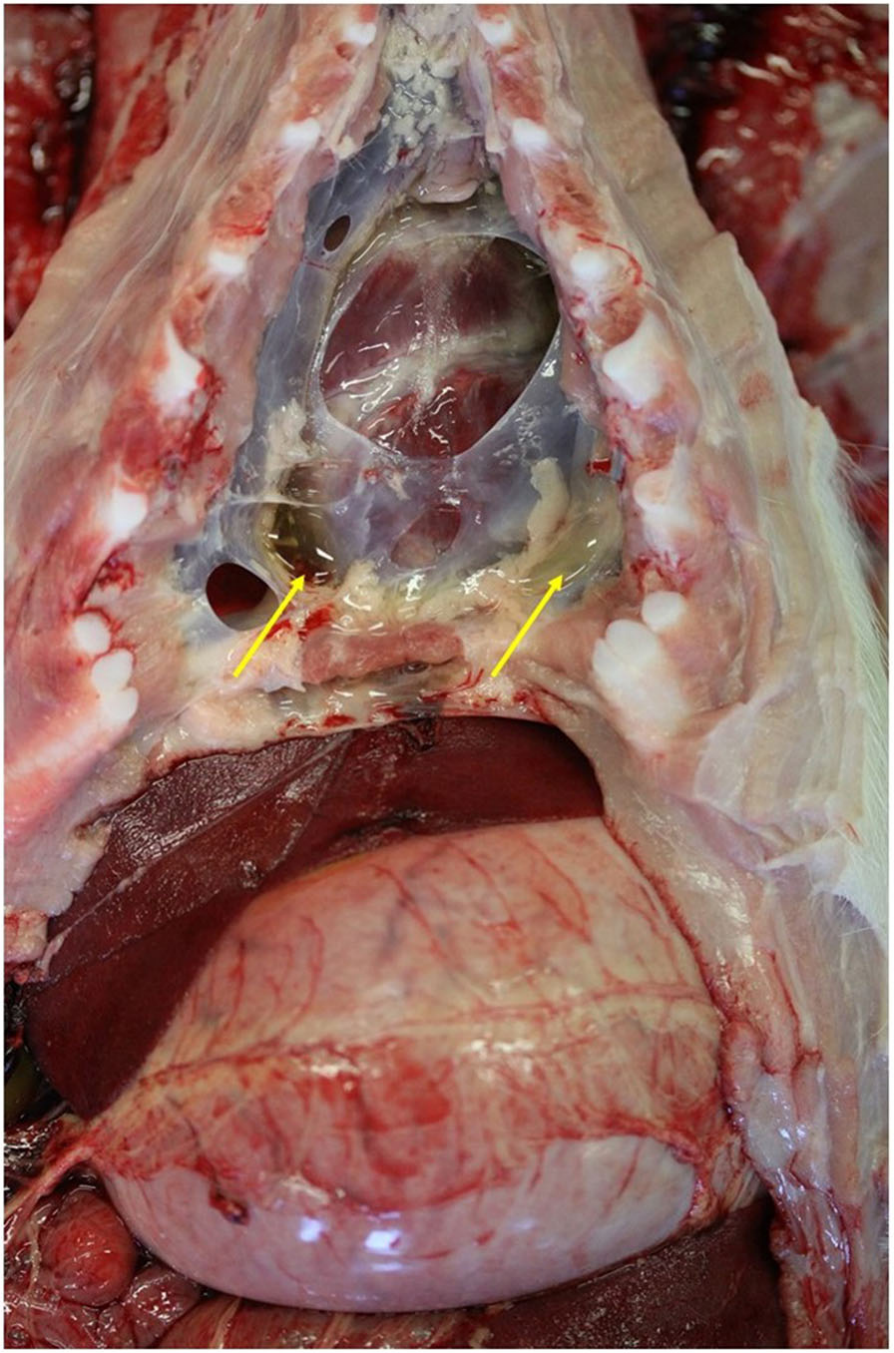
Transudate in the pleural cavity

**Fig. 2:**
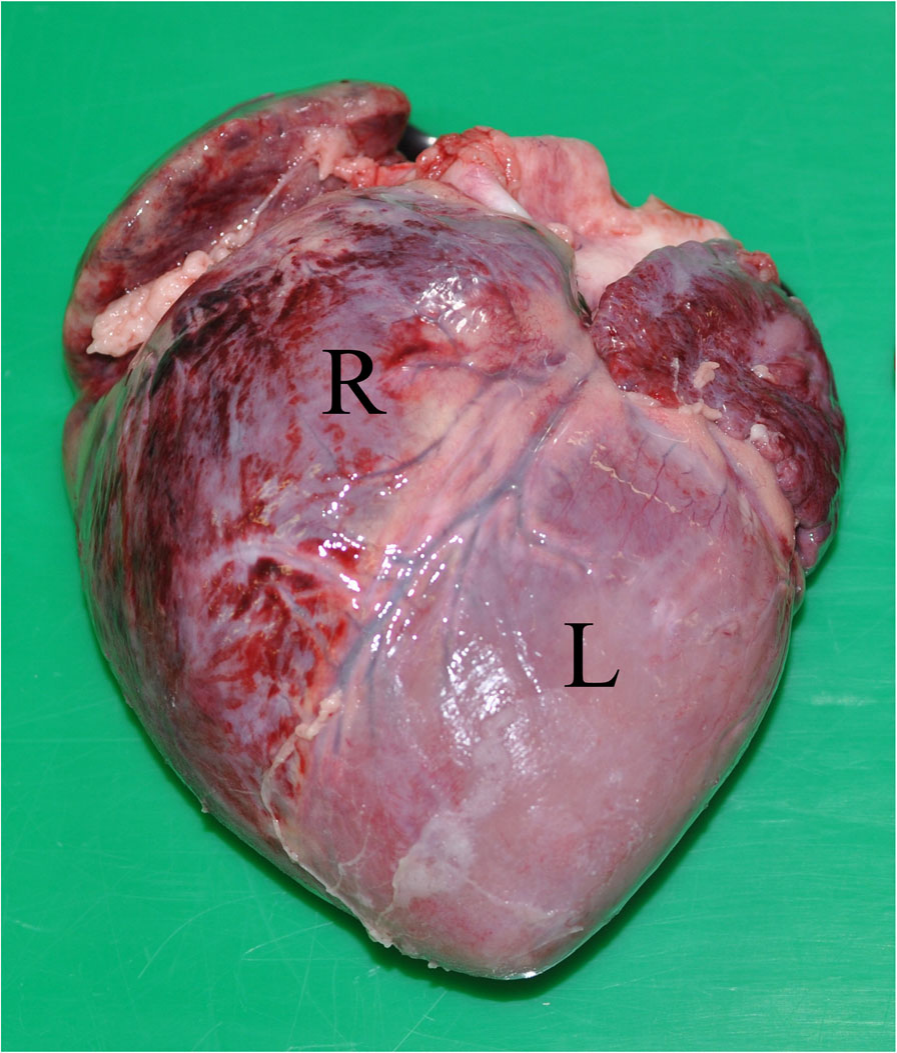
Subepicardial hemorrhages

MHD cases and control pigs underwent complete post mortem examinations. Pigs included as MHD cases had died suddenly and showed both typical macroscopic (Fig. 1) and (Fig. 2) microscopic lesions. Control pigs were selected from two of the farms submitting MHD cases. These pigs had the same genetic background and similar size as the confirmed MHD cases. Control animals were pigs showing good average daily gain compared to their littermates and other litters of the same batch, and they had not been treated due to sickness. Control animals showed no signs of pathological lesions when undergoing post mortem examinations.

A sampling protocol was elaborated to ensure a standardized sampling procedure for each organ. Tissue samples from the cardiovascular, digestive, immune, endocrine, integumentary, nervous, muscular, reproductive, respiratory and urinary systems were obtained (Table 3). Samples were dried with paper sheets to remove surplus blood. Intestinal samples were rinsed with water to remove intestinal contents prior to drying off with paper.

**Table 3:** Se concentrations in different organ samples from cases of Mulberry Heart Disease (MHD) and controls (mg/ kg dry weight)

Organ system	Organ samples	MHD		Control		*P*-value
		Mean Se (mg/ kg)	n	Mean Se (mg/ kg)	n	
Cardiovascular system	Myocardium, left ventricle	0.84 (0.15)	8	1.13 (0.12)	3	0.0070
	Myocardium, right ventricle	0.92 (0.04)	5	1.11 (0.15)	3	0.032
	Myocardium, septum	0.88 (0.04)	6	1.16 (0.06)	3	0.0059
Digestive system	Caecum	0.63 (0.11)	8	0.87 (0.18)	3	0.020
	Colon	0.77 (0.19)	8	1,04 (0.23)	3	ns
	Duodenum	0.78 (0.15)	7	1.20 (0.10)	3	0.0020
	Gastric ventricle	0.64 (0.13)	6	0.93 (0.08)	3	0.0090
	Ileum	0.86 (0.11)	8	1.04 (0.20)	3	ns
	Jejunum	0.90 (0.14)	8	1.27 (0.15)	3	0.0040
	Liver	1.29 (0.20)	8	1.77 (0.40)	3	0.020
	Pancreas	1.01 (0.28)	7	1.18 (0.57)	3	ns
Immune and endocrine system	*Ln ileocolici*	1.07 (0.42)	8	1.43 (0.16)	3	ns
	*Ln poplitei*	0.78 (0.27)	8	0.70 (0.13)	3	ns
	Parathyroid gland	0.88 (0.33)	5	0.80 (0.16)	3	ns
	Spleen	1.18 (0.17)	5	1.33 (0.06)	3	ns
	Thymus	0.97 (0.06)	8	1.16 (0.15)	3	0.012
	Thyroid gland	0.64 (0.13)	7	0.79 (0.12)	3	ns
	Adrenal gland	1.20 (0.08)	5	1.20 (0.10)	3	ns
Integumentary	Claw	0.41 (0.12)	5	0.66 (0.25)	3	ns
	Skin	0.14 (0.02)	7	0.25 (0.07)	3	0.0041
Muscular system	Diaphragm	0.39 (0.10)	6	0.62 (0.11)	3	0.020
	*M. biceps brachii*	0.35 (0.09)	8	0.64 (0.17)	3	0.0040
	*M. extensor carpi radialis*	0.34 (0.07)	8	0.66 (0.19)	3	0.0020
	*M. extensor digitorum longus*	0.33 (0.07)	8	0.65 (0.17)	3	0.0010
	*M. longissimus dorsi lumbalis*	0.35 (0.05)	5	0.62 (0.24)	3	0.042
	*M. longissimus dorsi thobaracis*	0.43 (0.23)	8	0.66 (0.25)	3	ns
	*M. psoas major*	0.34 (0.08)	8	0.67 (0.21)	3	0.0028
	*M. quadriceps femoris*	0.33 (0.08)	7	0.68 (0.22)	3	0.0040
	*M. semimembranosus*	0.32 (0.08)	8	0.64 (0.18)	3	0.0018
	*M. semitendinosus*	0.28 (0.05)	8	0.62 (0.20)	3	0.0011
Respiratory system	Lung	0.96 (0.23)	8	1.23 (0.06)	3	ns
Urinary system	Kidney	4.93 (0.57)	7	6.23 (1.07)	3	0.030

Se concentrations in internal and external samples from MHD and control pigs. Results are based on dry weight analyses

From all animals, tissue samples from the myocardium were examined histologically. Additionally, samples from tissues showing lesions were examined. Tissue samples of approximately 15 × 10 × 5 mm were fixed in 4% formaldehyde for one week and then dehydrated in graded ethanols and paraffin embedded. Sections (4 μm) were mounted on slides and stained with hematoxylin and eosin (HE). Light microscopic examination was conducted to specifically assess for myocardial lesions compatible with MHD. Pigs with characteristic macroscopic lesions combined with the following myocardial histopathology were considered MHD cases: Severe subepicardial and myocardial hemorrhages, swollen cardiac myofibers with loss of cross striations, and hypereosinophilic myofibers with pyknotic nuclei.

Samples of different tissues were collected at the same localization from each animal, both MHD cases and controls. Approximately 50 mg of tissue for ICP-MS analysis were placed in 1.8 mL cryotubes (Nunc Cryotube™, Sigma-Aldrich, Leirdal, Norway) and stored at − 20 °C until ICP-MS analysis. From the cardiac muscle, transmural samples were obtained from the center of the right and left free ventricular wall. Additionally, a transmural sample from the center of the septum was obtained. The gastric ventricle was sampled at the *Curvatura major*. Samples from the gastrointestinal tract were isolated from the proximal section of each intestinal region. From the liver, tissue was sampled from the lobe adjacent to the gallbladder. The splenic lobe of the pancreas was sampled. Both parathyroid glands were collected. Samples from the spleen, thymus, thyroid gland, skeletal muscles and kidney were obtained from the center of the organs. The lung was sampled from the right or left caudal lobes. The skin and bristles were sampled from the left or right lateral abdominal flank. Claw samples were from the distal tip of the left or right hoof wall.

A total of five to eight samples per tissue were analyzed for their total Se concentration (MHD cases) and compared to Se levels in samples from the three control animals. The concentrations of Se in collected organs were determined using inductively coupled plasma mass spectrometry (Agilent 8800 QQQ ICP-MS, Japan) at the Norwegian University of Life Sciences (NMBU/MINA). The organs were weighed, freeze-dried, transferred to acid cleaned Teflon tubes, and then weighed once more. The samples were added 2 ml water, 60 ng 74Se (enriched to 99.9%) as internal standard and 1.5 mL conc HNO3 (ultrapure quality). The samples, CRM and blanks were digested in an UltraClave and/or UltraWave from Milestone at 260 °C for about 20 min. After digestion, all samples were diluted to 15 ml prior to measurements [22]. Bovine Liver 1577c served as CRM. Limit of detection (LOD) and limit of quantification (LOQ) were calculated, 3 and 10 times respectively the standard deviation of the method blanks. In the present work LOD 0.005 mg Se/kg, LOQ 0.016 mg Se/kg.

Microbiologic examination was conducted on selected tissues from all animals. Specimens were inoculated on sheep blood agar for 48 h at 37 °C and 5% CO_2_. No bacterial growth was identified from any of the specimens tested.

All data were exported to Excel (Microsoft Corporation, Redmond, Washington) and then imported into JMP® Pro 14.0.0 (SAS Institute Inc., Cary, NC 2751, USA) for statistical analyses. Normality of data was tested by the Shapiro-Wilk test and homogeneity of variance. Differences between groups were analyzed by 1-way analysis of variance with concentrations of Se as the dependent variable and the animals’ status (MHD or control) as the independent variable. The farm, feed, sex and age were used as covariates. The p-level was set to 0.05. Potential outliers were assessed graphically and model diagnostics were performed.

**Fig. 3:**
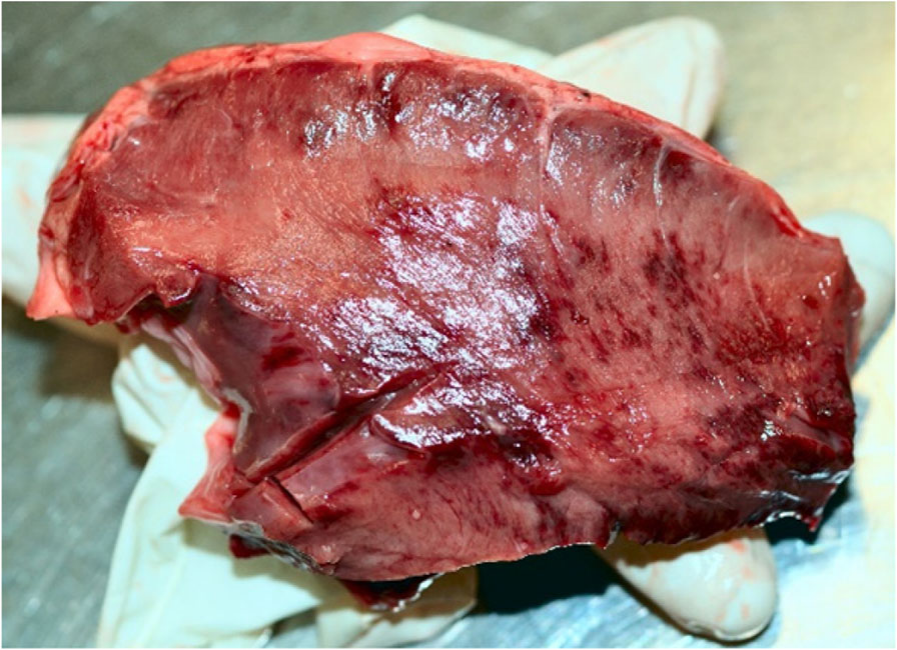
Myocardial hemorrhages

**Fig. 4:**
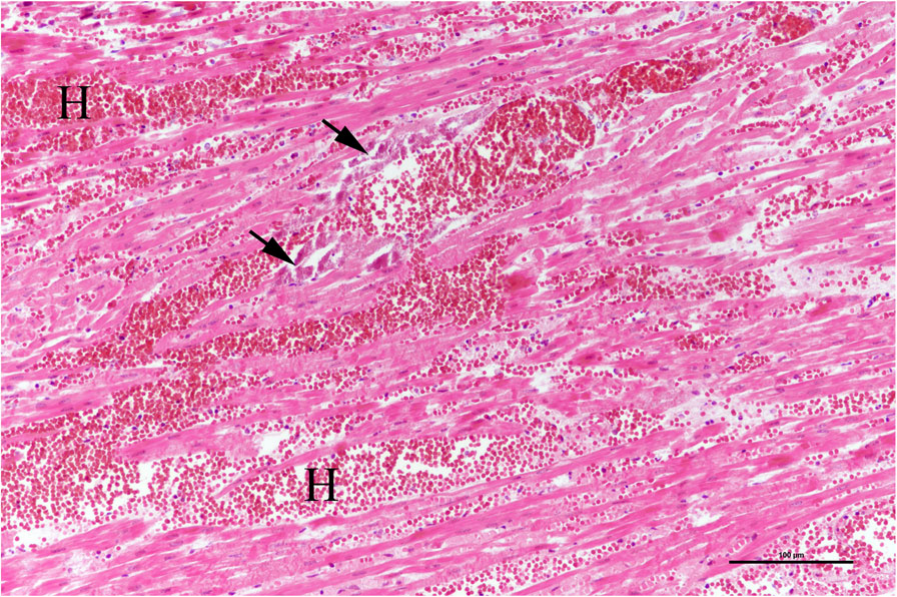
Interstitial hemorrhages

In all MHD cases, the diagnosis was confirmed by typical macroscopic lesions and histopathological findings. All MHD cases had straw-colored transudate in the pleural and pericardial cavity, often with fibrin strands (Fig. 1). Additionally, pulmonary edema as well as pale and reddened areas of the myocardium due to subepicardial (Fig. 2) and myocardial hemorrhages (Fig. 3) were found. Microscopically, interstitial hemorrhage was observed along with swollen cardiac myofibers that had lost cross striations (Fig. 4). Some histological sections also showed microthrombi, degenerative and necrotic areas with local mineralization.

No lesions were found in skeletal muscles or liver tissues, except MHD case no. 8, also showing macro- and microscopic lesions compatible with *Hepatosis dietetica*.

Based on all ten skeletal muscular samples, mean Se concentrations in MHD cases were 0.34 (0.01) mg/ kg compared with 0.65 (0.02) mg/ kg in control pigs (*P* < 0.0001). Samples from the cardiovascular system showed significantly lower mean Se concentrations in all sampled areas of MHD cases compared with control pigs. Greater difference in mean Se concentrations was observed in the left ventricular wall of the heart; 0.84 (0.15) mg/ kg vs. 1.13 (0.12) mg/ kg (*P* = 0.0070, Table 3). If combining the results from the three cardiac samples, the mean Se concentration in MHD cases was 0.87 (0.02) mg/ kg as opposed to 1.12 (0.04) mg/ kg (*P* < 0.0001) in the cardiac samples from controls.

Within the digestive system, lower average Se concentrations were found in samples from the MHD cases’ gastric ventricle, duodenum, jejunum and caecum. In the liver samples, mean Se concentrations of MHD cases were 1.29 (0.20) mg/ kg vs 1.77 mg/kg (0.40) in controls (*P* = 0.020). No differences between MHD cases and controls were found in samples from the adrenal glands, claws, colon, ileum, lungs, lymph nodes, pancreas, parathyroid gland, spleen or thyroid gland.

## Discussion and conclusions

The sampled pigs in this report constitute a heterogenous group since multiple genetic lines fed different feed and pigs with differing bodyweight are represented. These are factors potentially influencing the pigs’ susceptibility to oxidative stress and MHD. We find it interesting that although our limited number of sampled pigs are different in terms of genetics, size and feeding regimes, the variation of Se concentrations in a given organ is low between MHD cases.

The results from this study provide information about the Se distribution both in internal and external organs in MHD cases and designates muscle tissue as particularly interesting regarding the diagnostic approach. Most reports describing pigs with MHD refer to vitamin E and Se concentrations in liver samples [10, 18–20, 23]. Although biodilution cannot be excluded (difference in weight and age), our results did render significant differences in liver Se concentrations between MHD cases and control animals. Mean liver concentrations of MHD cases (dry basis) in this study were 1.29 mg/kg (0.20). A liver concentration of 1.2 mg Se/ kg (dry basis) was suggested by Lindberg and Siren as normal [24]. Other studies have stated that MHD cases apparently show Se liver concentrations within what is considered the normal range [10, 19]. Thus, the questions arise if the Se concentrations in liver samples from healthy, high-yielding pigs of today should actually be higher, if other samples should be collected and analyzed, and if genetic differences between breeds could affect the actual Se requirements. A recent study has shed light on the micronutrient-genetic relationships and showed that genetic background can affect the intake of minerals [25]. Typically MHD cases are pigs growing rapidly. This feature combined with possible individual disparities regarding feed intake and efficiency may contribute to a disruption in proper mineral intake and cause certain individuals within a group of pigs to succumb to MHD.

Most previous reports on MHD also include Se concentrations in myocardial samples, which is reasonable since pathological findings are found invariably in the myocardium of MHD cases. Here, a significant difference was found between MHD samples and controls with respect to the samples isolated from the septum (*P* < 0.01), left (P < 0.01) and right ventricle (*P* < 0.05) of the myocardium. This is not in accordance with previous studies, reporting *no* difference between MHD cases and controls in terms of Se concentrations in cardiac samples [18, 26]. In a recent publication the authors stated that different muscle tissues have distinct intrinsic mitochondrial respiratory functions, which likely influences the efficiency of oxidative phosphorylation and could potentially alter reactive oxygen species (ROS) production [27] .The cardiac muscle with its high metabolic demand is rich in mitochondria, accounting for approximately 35% of the cardiac tissue volume. Skeletal muscles exhibit approximately half of the mitochondrial density found in the cardiac muscle. Since mitochondria are the most important cellular source of ROS [28], this may contribute to the cardiac muscle susceptibility to ROS-induced oxidative injury if a pig is deficient in antioxidant factors. A rodent Se deficiency and repletion model showed a distinct distribution of selenoenzymes, suggesting that the heart may be the organ most sensitive to oxidative stress [29]. The fact that all MHD cases showed macroscopic and microscopic lesions in cardiac samples but not in skeletal muscles may support this theory. Within the cardiac muscle, some areas may be of particular importance for sampling, like the papillary muscles. They are located in both ventricles of the heart and it has been shown that papillary muscles are prone to fibrosis upon oxidative stress insults [30]. A more standardized approach for sampling of the cardiac muscle of MHD cases may contribute to less interindividual variability regarding Se concentrations.

In industrialized pork production, feed costs account for approximately 60 to 70% of the total production costs [16, 17, 31, 32]. Since higher economic outputs of pork production can be achieved by improved feed efficiency, selection for high lean growth rate and reduced backfat, commercial pig lines have been systematically bred over decades to improve these traits [15, 33, 34]. Norsvin is a breeding company owned by Norwegian pig producers with a research department at the Norwegian University of Life Sciences (NMBU). The breeding program pursued by Norsvin has e.g. led to a reduction in necessary feed per kg of weight gain and increased average daily gain (ADG) in weaned pigs and grower-finishers over the last ten years [35, 36]. In 2007, Norwegian conventional weaned pigs (typically DLYL, LLLL or YYLL) between approximately 10 and 30 kg live weight (LW) showed ADG of 489 g vs. 582 g in 2017. Grower-finishers between approximately 30 kg and 115 kg LW showed an ADG of 955 g in 2007 vs. 1018 g in 2017 (Table 4). During the same time period (2007 to 2017), the amount of feed per unit gain was reduced in both weaned pigs (1.81 to 1.71) and grower-finishers (2.74 to 2.68). The lean meat percentage in finisher pigs rose from 56.5 to 59.8%. Typically, Norwegian pig feed is added between 0.35 and 0.40 mg Se/ FU, both for weaned and grower-finisher pigs (until recently the dominating Se source has been inorganic sodium selenite). This means that during the last decade, there has been a reduction in available Se. This is due to the reduced amount of necessary feed per unit weight gain and the concurrent increase in body protein deposition, possibly contributing to the occurrence of MHD.

**Table 4:** Development of Norwegian pig production results from 2007 to 2017

Production trait	Age group	Year	
		2007	2017
Average daily weight gain (g/ day)	Weaned pigs	489	582
	Grower-finisher pigs	955	1018
Feed units per kg gain (FU/ kg)	Weaned pigs	1.81	1.71
	Grower-finisher pigs	2.74	2.68
Lean meat percentage (%)	Grower-finisher pigs	56.50	59.80

The numbers origin from the annual Ingris report, an online tool available for both Norwegian breeding (nucleus and multiplier) herds as well as piglet producing and finisher units. In 2007, 35% of sow farms and 3.1% of finisher farms were represented in the Ingris annual report. In 2017, 54% of sow farms and 12.3% of finisher farms were represented. In the Norwegian pig production system, weaned pigs are between approximately 10 kg LW and 30 kg LW. Grower-finishers are between approximately 30 kg LW and 115 kg LW

Several authors have considered the theory of resource allocation in lean and feed efficient pigs, which is built on the assumption that different biological processes require different nutritional resources [37–40]. The systematic selection for leaner pigs over decades may have led to a trade-off towards growth in situations where nutritional resources are scarce. Processes involving e.g. the immune and antioxidative status of the animal can thereby be weakened and result in pigs more susceptible to oxidative assaults. Our results showing significantly lower Se concentrations in samples from the thymus of MHD cases may strengthen this theory.

The MHD cases showed significantly lower mean Se concentrations in the samples from the gastric ventricle (*P* < 0.01), the duodenum (*P* < 0.005), jejunum (*P* < 0.005) and caecum (*P* < 0.05). These findings may partly be explained by the absorption pattern of Se in the intestinal tract. In swine, more Se is absorbed in the last part of the small intestine, cecum and colon than in the stomach and proximal parts of the small intestine [41]. Oxidative stress is a major cause of gastrointestinal (GI) damage [42] and robust immunologic mechanisms are required to protect the mucosal surface. The Se dependent glutathione peroxidase GSHPx-GI appears to be the major glutathione-dependent peroxidase in the GI tract and this molecule could play a major role in protecting mammals from the toxicity of ingested lipid hydroperoxides [43, 44]. Se dependent glutathione peroxidase is expressed at lower concentrations when the organism enters Se deficiency [45]. A possible explanation to our Se measurements in the proximal parts of the GI tract may therefore be a lower absorption of Se in proximal intestinal segments combined with lower expression of GSHPx-GI in MHD cases than controls. Less difference in Se levels between MHD cases and controls were found in caecum and colon. Significantly lower mean Se concentrations in skin (*P* < 0.005) and kidney (*P* < 0.05) samples from MHD cases were found, probably due to lowered selenoprotein expression during Se deficiency [46, 47].

No vitamin E analyses were included in this study due to budget limitations. Previous results have indicated that pigs with MHD have lower tissue alpha-tocopherol concentrations than the control pigs [26], and therefore its role in the pathogenesis of MHD needs further elucidation [2]. The trace elements calcium, copper, zinc, magnesium, iron would have been interesting to add to the analyses, since it has previously been described that Se deficiency can alter the distribution of other minerals [48, 49].

In other animals, like cattle, the Se requirements are differentiated according to muscularity [50]. The question whether lean, feed efficient pigs have higher Se requirements than slower growing pigs with a lower lean meat percentage is indeed worthwhile pursuing.

In conclusion, this study shows discrepancies in tissue Se concentrations between MHD cases and controls. As mentioned initially, the common practice regarding collection of samples from MHD cases in the field could be extended to include samples from the diaphragm or skeletal muscles for Se analysis, since these organ samples are easy accessible during field necropsy. Although the variation in Se concentrations of a given organ between MHD cases of different genetic origin and age was low, this report includes a limited number of pigs. Therefore our results should be affirmed by a larger randomized controlled trial.

## Data Availability

The data generated during the current case report are kept and stored by the corresponding author. The data are available from the corresponding author on reasonable request.

## Abbreviations

(ICP-MS): Inductively coupled plasma mass spectrometry
DM: Dry matter
GSHPx-GI: Intestinal form of glutathione peroxidase
HE: Hematoxylin and eosin
MHD: Mulberry Heart Disease
Q: Quadropoles
Se: Selenium
Te: Tellurium
